# Potential use of dry powder of *Vossia cuspidata* (Roxb.) Griff. rhizomes and leaves in methylene blue dye remediation

**DOI:** 10.1038/s41598-023-37987-0

**Published:** 2023-07-08

**Authors:** Hossam E. A. Awad, Ahmad M. Mohammad, Emad A. Farahat

**Affiliations:** 1 Botany and Microbiology Department, Cairo University, Giza, 12613 Egypt; 2 Chemistry Department, Faculty of Science, Cairo University, Cairo, 12613 Egypt; 3 Botany and Microbiology Department, Helwan University, Cairo, 11795 Egypt

**Keywords:** Plant sciences, Environmental sciences, Chemistry

## Abstract

Phytoremediation is a promising, cost-effective, and eco-friendly process for wastewater treatment. Herein, the dry biomasses of *Vossia cuspidata* (Roxb.) Griff. leaves (PL) and rhizomes including aerial stems (PR) were used to effectively remediate methylene blue (MB) dyes. Interestingly, the adsorption uptake and removal efficiency of MB by PR were higher than those of PL; exceeding 97 and 91% in 35 and 25 min for 0.1 and 0.4 g/L MB, respectively. The MB diffusion within the PL and PR was insignificant and the adsorption kinetics was principally controlled by the surface MB–adsorbent interaction, as consistently approved by the pseudo-second order kinetic model. In addition, the adsorption increased rapidly with the plant dosage with high dependence on the initial MB concentration. Moreover, the impact of shaking speed on the adsorption was minor but temperature played a critical role where the highest efficiencies were recorded at 30 and 40 °C on PL (91.9%) and PR (93.3%), respectively. The best removal efficiencies were attained with PR at pH 6, but with PL at pH 8. The Temkin isotherm could perfectly simulate the experimental data (R^2^ > 0.97); suggesting a linear decrease of the adsorption heat of MB with the plant coverage.

## Introduction

According to United Nations World Water Development Report in 2020, around 4 billion people are subjected to severe physical water scarcity for at least 1 month in a year^1,2^. This number is expected to increase rapidly with the population growth to reach up to 10.2 billion people in 2050^3^. In another estimate by the Food and Agriculture Organization (FAO) of the United Nations, 3.2 billion people live in agricultural areas with high to very high water shortages or scarcity, of whom 1.2 billion people—roughly one-sixth of the world’s population—live in severely water-constrained agricultural areas^4^. Hence, various plans should be proposed to remediate wastewater, particularly from sewage plants. Environmental pollution by dyestuff is a global issue that needs more attention for a healthy environment. Pollution with dyes causes hazardous impacts for all living organisms and depending on the ecosystem, they may further affect photosynthesis and food chains^5^. Typically, dyes are extremely toxic and carcinogenic, together with persisting natural degradations^6^. Unfortunately, these pollutants are widely used in considerable amounts in several textiles, cosmetics, food, pharmaceutical, paper, and leather industrial activities. The world’s annual production of dyes is more than 700 thousand tons. About 2% of them are directly discharged from manufacturing plants into effluents, and roughly 10% of dyes are lost throughout the dyeing industries process^7–10^. The discharge of these dyes in the ecosystem causes undesirable environmental loads^11–14^.

Methylene blue (MB, also known as methylthioninium chloride) is one of the most common substances that is widely used in dyeing industries. The presence of this dye even in low concentrations causes undesirable coloration of the aquatic ecosystem. Removal of (MB) using some natural raw materials such as rice husk, hair, and guava seeds was studied by several groups^15,16^. However, the management of this pollutant is still a growing issue, necessitating the development of new technologies, appealing to the ecological valorization concerns^17^. Among the recent methods for the decolorization of dye-containing effluents, the removal via adsorption onto specific adsorbents appeared highly promising from a considerable environmental perspective^18^. Despite the wide variety of sorbents in the literature, biomass derived from agricultural waste or plant residues appears highly attractive for the adsorption of emerging inorganic heavy metals and organic dyes contaminants^19^. Generally, biomass is cost-effective, environmentally benign and renewable biosorbent of high contents of carbon and cellulose, and diverse functionality^20^. This diversity is accustomed to biomass due to the availability of multiple surface functional –OH, –C=O and –COOH groups that furnish a high adsorption tendency to a large scale of adsorbates^21,22^. The role of activation of biosorbents and their applications to remediate wastewater from heavy metals and dyes were recently reviewed^2,19^.

Generally, in phytoremediation where biomass from plant residues is used to remediate contaminated industrial streams, the same principles and mechanistic steps of adsorption were applied. This involved a cascade of simultaneous events transporting the contaminant molecules from the bulk solution to the interface by migration, diffusion, and/or convection before getting adsorbed at the solid/H_2_O interface^23^. A structural modification of the adsorbate at the solid/H_2_O interface or within the pores of the adsorbent for better packing with lateral interactions of adsorbate molecules is possible^23^. In natural ecosystems, adsorption competition between molecules of different natures and molecular weights should be additionally considered^24^. Finally, the surface interaction (whether physically or chemically) of the adsorbate at the adsorbent surface and the possible desorption should, moreover, be addressed. Any of these steps may determine the kinetics of the adsorption process, and accordingly, several kinetics models and adsorption isotherms were developed^25–28^.

Numerous studies have been conducted on the use of plants and their biomass in the cleanup of contaminated environments^29–34^. More than 400 plants including many hydrophytes have been assigned as prospective phytoremediators^35–38^. For example, *Neolamarckia cadamba* leaves succeeded to remediate MB dye and to decrease significantly the total dissolved solids (~ 27%), conductivity (~ 21%), hardness (~ 89%) and chemical oxygen demand (~ 66%) of the sewage belts of Yamuna River in Allahabad, Uttar Pradesh, India^39^. Alternatively, the aquatic plant, *Azolla pinnata* as a biosorbent prospered to impart ~ 85% decolorization of MB dye (25 mg/L) from contaminated wastewater streams within 48 h^40^.

*Vossia cuspidata* (Roxb.) Griff. **(**family Poaceae) is a long-lived macrophyte grass that is widely grown in Southeast Asia and Tropical Africa^41^ The natural vegetation of the River Nile in Egypt has drastically decreased because of its recent invasion of the aquatic environment^42^. In Egypt, *V. cuspidata* was investigated for its ability to phytostabilize heavy metals from polluted water canal^43–46^. They reported that this species can accumulate significant levels of Cd, Cu, Mn, Pb, Ni, Co, and Zn ions in their roots. However, to our knowledge, there are no data available about the use of dry biomass of *V. cuspidata* in the phytoremediation of industrial MB dyes. Therefore, the objective of this study is to investigate the potential use of the dry biomass of *V. cuspidata* in the phytoremediation of textile MB dyes. The removal of MB dye was employed for marking the adsorption efficiency and capacity of the biomass of the *V. cuspidata* plant leaves and stems including the underground rhizomes and aerial stems.

## Materials and methods

### Preparation of the adsorbent materials

The plant materials were collected from the natural habitats of *V. cuspidata* along the Nile River, Cairo, Egypt. Plant samples were transported to the laboratory and divided into rhizomes, aerial stems, and leaves. We refer to the *V. cuspidata* plant’s rhizomes and stems as PR, and plant leaves as PL. After separation, all organs were washed by running tap water several times to clean the plant from debris and dust, then washed with distilled water. Each plant organ's component was first air-dried, followed by oven drying at 60 °C for 72 h to achieve a constant mass, and finally ground to a fine powder in an agate mortar and sieved through a 0.2 mm mesh sieve. The dry biomass of PL and PR was used as an adsorbent for MB dye’s remediation. The functional groups of the adsorbent were characterized by Fourier Transform Infrared spectroscopy (FTIR, Model Bomen/MB102, ABB Company, Switzerland). The surface morphology of the *V. cuspidata* was evaluated by the field-emission scanning electron microscopy (FE-SEM, Quanta FEG250).

### Adsorption studies

To test the ability of dry biomass of the selected organs of *V. cuspidata* to remediate the MB dye, several tests were conducted including the effect of contact time, initial adsorbate concentration and adsorbent mass, temperature, pH, and shaking speed. During the adsorption studies, the removal efficiency of MB onto the PL and PR was calculated using Eq. (1)^47^:

1 $$Removal \,(\%)=\left[1-\left(\frac{{C}_{e}}{{C}_{0}}\right)\right]\times 100,$$

where ${C}_{e}$ (mg/L) is the final (equilibrium) MB dye (adsorbate) concentration and ${C}_{0}$ (mg/L) is the initial MB dye concentration in the bulk solution. All adsorption measurements were repeated three times before testing the curve fitting, calculating the standard error, and performing the statistical analysis.

#### Effect of contact time

1 g dry biomass of PL or PR was mixed with 100 mL of 0.1, 0.2, 0.3, and 0.4 g/L MB solution. Each solution was represented by three replicates to avoid a decrease in the pollutant’s volume. The solutions were kept at room temperature and natural pH. Supernatant samples of 10 mL were taken every 5 min and centrifuged then the remaining dye was analyzed at 664 nm using a spectrophotometer (PerkinElmer Lamda 25 UV/VIS spectrophotometer). The MB dye absorption at the same wavelength was calibrated.

To elucidate the adsorption kinetics, the amount (mg) of MB dye adsorbed by the dry plant biomass at a given time (${q}_{t}$) and at equilibrium (${q}_{e}$) were calculated according to Eqs. (2) and (3), respectively^48^:

2 $${q}_{t}=\frac{\left({C}_{0}-{C}_{t}\right)\times V}{W},$$

3 $${q}_{e}=\frac{\left({C}_{0}-{C}_{e}\right)\times V}{W},$$

where ${C}_{t}$ (mg/L) is the concentration of MB dye (adsorbate) at a given time in the bulk solution of a volume $V$ (L). The mass of PL or PR biomass (adsorbent) is $W$ (g).

The kinetics data of MB adsorption on the PL or PR biomass were tested for fitting in the pseudo-first-order (PFO), pseudo-second-order (PSO), and Weber and Morris (WM) intraparticle diffusion models^48–50^. According to the PFO rate model Eq. (4) and its linearized form Eq. (5) that was developed by Lagergren, ${q}_{t}$ increases exponentially with time, as following^49,51^:

4 $${q}_{t}={q}_{e}\left[1-{e}^{-{k}_{1}t}\right],$$

5 $$\mathrm{ln}\left({q}_{e}-{q}_{t}\right)=\mathrm{ln}{q}_{e}-{k}_{1}t,$$

where ${k}_{1}$ (min^−1^) is the first-order rate constant.

On the other hand, the PSO model proposes the following empirical linear formula Eq. (6) for ${q}_{t}$ vs. time^52,53^:

6 $$\frac{t}{{q}_{t}}=\frac{t}{{q}_{e}}+\frac{1}{{{{k}_{2}q}_{e}}^{2}},$$

where and ${k}_{2}$ (mg/g/min) is the second order rate constant

The initial rate of adsorption ($h$) can be assessed according to the PSO model as follows Eq. (7)^52,53^;

7 $$h={{{k}_{2}q}_{e}}^{2}.$$

Alternatively, the WM model suggested a linear variation of ${q}_{t}$ with the square root of time according to Eq. (8)^48,50^:

8 $${q}_{t}={k}_{3}{t}^{1/2}+C,$$

where ${k}_{3}$(mg/g/min^0.5^) and $C$ (mg/g) stand, respectively, for the rate and correlation constants of the intraparticle diffusion model.

#### Effect of initial adsorbate concentration

Five different adsorbate concentrations (0.025, 0.05, 0.1, 0.2, and 0.4 g/L) of MB were prepared. In three separate reactors, 100 mL of each concentration of MB dye was mixed with one of these three loadings (1, 2, and 3 g) of PL and PR. The solutions were kept at room temperature and natural pH (≈ 7) to the desired contact time and then analyzed.

#### Effect of adsorbent dry biomass

Six different loadings (0.5, 1, 1.5, 2, 2.5, and 3 g) of PL and PR dry biomass were mixed with 100 mL of 0.1, 0.2, 0.3, and 0.4 g/L MB solution, and solutions were kept at room temperature and natural pH (≈ 7). Supernatant samples of 10 mL were taken for analysis at the optimal contact time for each plant material.

#### Effect of temperature

Flasks containing 100 mL of 0.1 g/L MB solution were placed in a water bath to adjust their temperature before mixing with 1, 2, or 3 g of each plant material. The solutions were then incubated at seven different temperatures (20, 25, 30, 35, 40, 45, and 50 °C) and natural pH (≈ 7) in a water bath. At the optimal contact time, 10 mL of supernatant was taken for analysis.

#### Effect of pH

100 mL of 0.1 g/L MB solution was treated with 1, 2, and 3 g of each plant material at five different pH (hydrogen ion) concentrations (6, 6.5, 7, 7.5, and 8, respectively). The tested solutions were adjusted to the desired pH value before mixing with the adsorbent using 0.1 M NaOH and 0.1 M HCl solutions. The mixtures were left to the equilibrium contact time before analysis.

#### Adsorption isotherm models

Four adsorption models; namely, the Freundlich, Langmuir, Temkin, and Dubinin–Radushkevich (D–R) isotherms were employed. The Freundlich model which is concerned with the adsorption onto heterogeneous surfaces assumed the following relationship Eq. (9) between ${q}_{e}$ and ${C}_{e}$^54–56^:

9 $$\mathrm{ln}{q}_{e}=\frac{1}{n}{\text{ln}}{C}_{e}+{\text{ln}}{k}_{F},$$

where ${k}_{F}$ is the adsorption capacity (L/mg) and *n* is an empirical constant inferring the relative distribution of energy and heterogeneity at the adsorbate surface^54^. For 1/*n* values between 0.1 < 1/*n* < 1, the adsorption process is favorable^54^.

On the other hand, the Langmuir isotherm that fitted perfectly for the adsorption of a monolayer of adsorbate onto the surface of the adsorbent assumed the uniformity of the adsorbent that ensures the equivalency of adsorption sites^57^. The adsorption of the adsorbate onto a finite number of identical active sites of the adsorbent with the same adsorption mechanism is also assumed. The interaction of the adsorbed molecules together, regardless of the surface coverage, is also forbidden^58–60^. Mathematically, the Langmuir adsorption isotherm model is represented by Eq. (10):

10 $$\frac{{C}_{e}}{{q}_{e}}=\frac{1}{{K}_{L}{q}_{m}}+\frac{{C}_{e}}{{q}_{m}},$$

where ${q}_{m}$ (mg/g) is the maximum adsorption capacity and ${K}_{L}$ (L/mg) is the Langmuir equilibrium constant that is related to the energy of adsorption^61^.

However, the Temkin model is expressed in the following formula Eq. (11); accounting for the possible indirect adsorbate/adsorbent interactions^48,56,62^:

11 $${q}_{e}=\left(\frac{RT}{b}\right){\text{ln}}A+\left(\frac{RT}{b}\right){\text{ln}}{C}_{e},$$

where $b$ (J/mol) and $A$ (L/g) are the Temkin constants, R is the gas constant (8.31 J/mol/K) and T (K) is the absolute temperature.

Finally, the D–R isotherm that realizes the surface's non-uniformity in accommodating different adsorption modes and fits perfectly for the pore-filling mechanism is expressed in the following Eqs. (12)–(14)^56,63,64^:

12 $${\text{ln}}{q}_{e}={\text{ln}}{q}_{m}-\beta {\varepsilon }^{2},$$

13 $$\varepsilon =RT{\text{ln}}\left(1+\frac{1}{{C}_{e}}\right),$$

14 $$E=\frac{1}{\sqrt{2\beta }},$$

where $\varepsilon$ is the Polanyi potential and $\beta$ (mol^2^/kJ^2^) is the D–R constant or activity coefficient that can directly be converted to the average adsorption energy, $E$, (kJ/mol).

#### Effect of shaking speed

100 mL of 0.1 g/L MB solution was mixed with (1, 2, and 3 g) of each plant material using five different shaking speeds (100, 150, 200, 250, and 300 rpm, respectively). The solutions were kept on the shakers at room temperature and natural pH (≈7) to the optimal contact time for each plant material, then moved to analysis.

### Ethical approval

Ethics approval in this study, plant materials were collected and used according to the national regulations. The utilization of these species for experimental purposes does not require any special permit. All methods in our study comply with relevant institutional, national, and international guidelines and legislation. The plant was identified by the last author according to Boulos^31^ and a voucher id (8105) for the plant was deposited at the herbarium of Helwan University.

## Results

### Materials characterization

**Figure 1:**
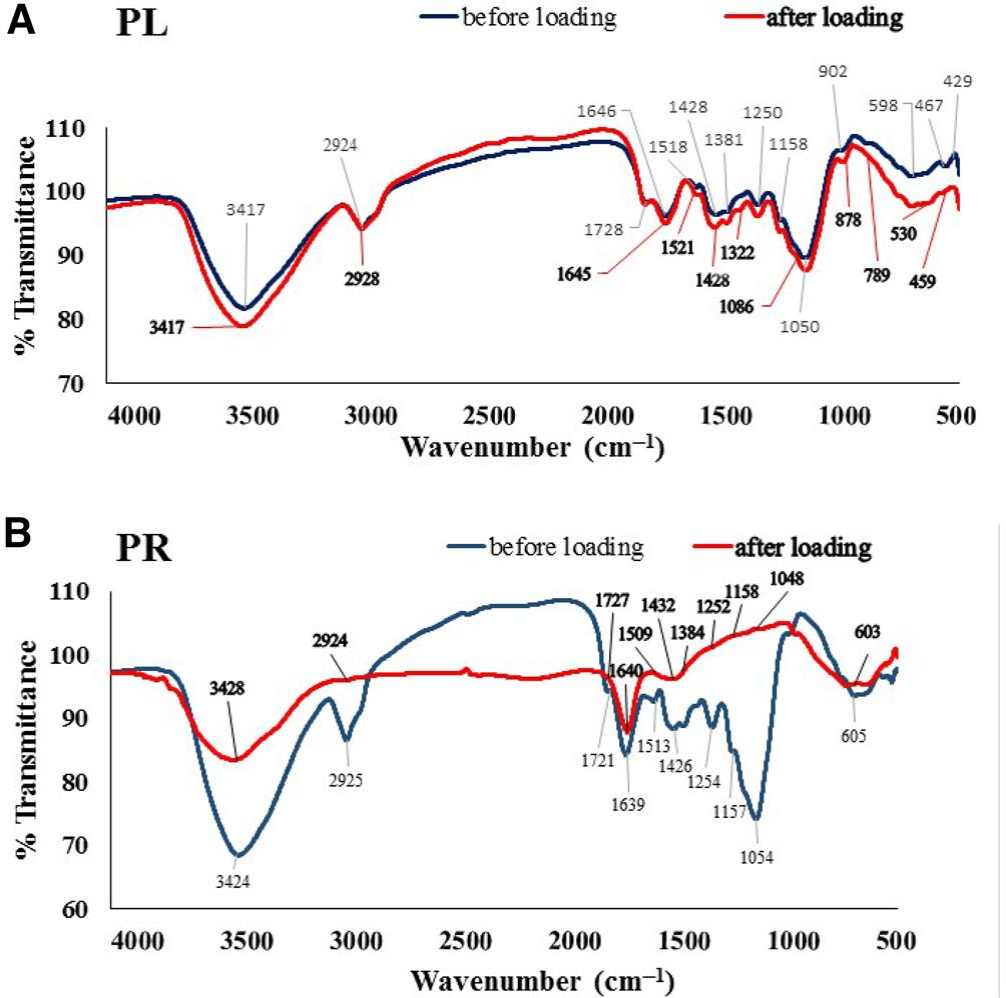
FTIR spectra of *V. cuspidata* leaves, PL (**A**) and rhizome, PR (**B**) powder before and after MB loading.

Previous chemical analysis verified and estimated the contents of Na, K, N, Mg, P, Ca, ether, crude fibres, proteins, ash, and carbohydrates with seasonable variations in the aboveground leaves and stem, and in the belowground roots and rhizome of *Vossia cuspidata*^65^. Figure 1A,B show the FTIR of the PL and PR biomasses before “pristine” and after getting loaded with the MB dye. As seen in Fig. 1, the raw plant materials owned major peaks at 3417, 2924, 1646, and 1050 cm^−1^ for PL (Fig. 1A), and at 3424, 2925, 1639, and 1054 cm^−1^ for PR (Fig. 1B). Other minor peaks appeared at 1728, 1646, 1518, 1428, 1381, 1250, 1158, 902, 598, 467, and 429 cm^−1^ for PL (Fig. 1A), and at 1721, 1513, 1426, 1254, 1157, and 605 cm^−1^ for PR (Fig. 1B). Loading of MB onto the PL and PR biomasses resulted in obvious shifts in the peaks positions (Fig. 1A,B), which can correlate certainly to modulations of the vibrational energy structure of PL and PR due to their interaction with the MB dye.

**Figure 2:**
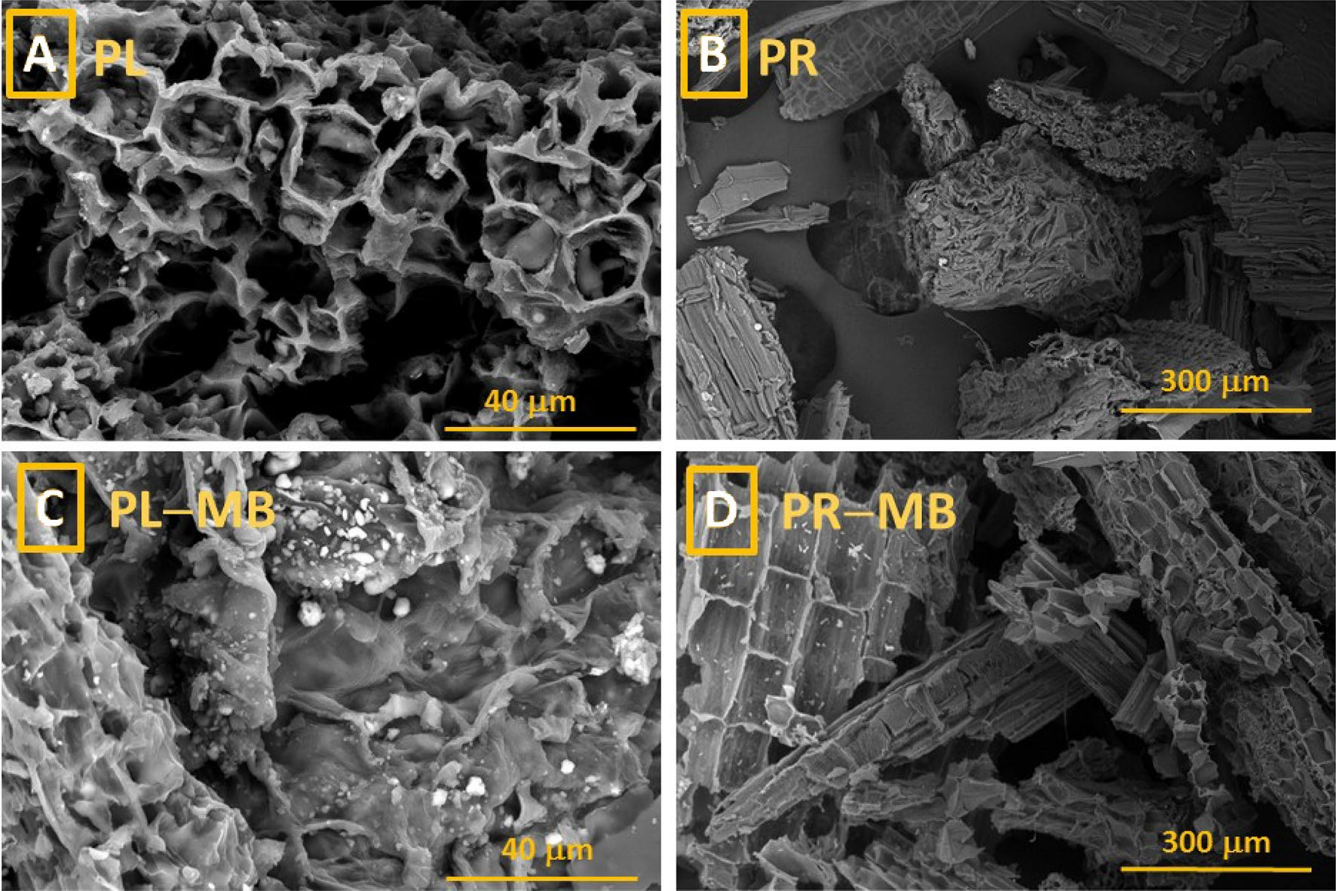
FE-SEM images of the *V. cuspidata* leaves (**A,C**) and rhizome (**B,D**) before (**A,B**) and after (**C,D**) loading with MB-contaminated solution.

The morphology of the *V. cuspidata* biomass before and after loading with MB was inspected. Figure 2A–D depicts the surface FE-SEM images of the PL (Fig. 2A,C) and PR (Fig. 2B,D) before and after soaking in MB-contaminated solution. As obviously seen in Fig. 2A,C, the PL displayed intensive microcavities at the surface with porous texture which is favorable for pore-filling adsorption mechanism. On the other hand, the PR biomass appeared as agglomerated tubular bundles. After loading with MB (Fig. 2C,D), the PL and PR surface appeared partially-coated with white debris.

### Adsorption kinetics

**Figure 3:**
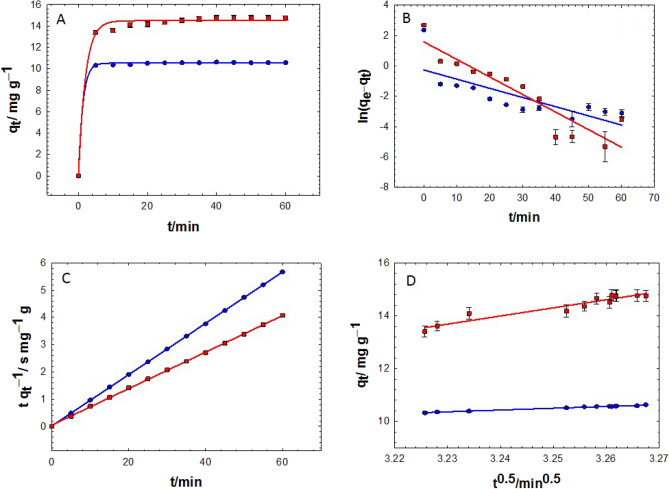
(**A**) Effect of contact time on the adsorption capacity of 0.1 g/L MB (natural pH) onto 1 g of PL (blue circles) and PR (red squares). The linear fitting of the experimental data was done by using the PFO (**B**), PSO (**C**), and WM (**D**) kinetic models.

The experimental data for the MB adsorption by the PL and PR dry biomass were fitted in the PFO Eqs. (4) and (5), PSO Eq. (6), and WM Eq. (8) kinetics models (Fig. 3A–D, Tables 1, 2). The regression coefficient, R^2^, was assigned as a fitting index to evaluate the suitability of the different models to the adsorption data. It was revealed that the adsorption of MB onto both PL and PR powders was very rapid as ${q}_{t}$ increased semi-exponentially over time, jumping to saturation within the first few minutes (ca. 5 min) of adsorption (Fig. 3A). The equilibrium adsorption uptake, ${q}_{e}$, of PR (14.50 mg/g) was higher than that of PL (10.54 mg/g). After 5 min of contact with MB, 1 g of PR powder showed 89 and 85% removal when treated with 0.1 g/L and 0.4 g/L MB, respectively. On the other hand, the PL powder amounted to removal efficiencies of 91 and 64% when treated with the same concentrations, respectively. Increasing the contact time showed a slower removal rate. The removal efficiencies of MB onto PR powder extended to 97 and 91% at 35 and 25 min, respectively, for the same abovementioned solutions. However, 94 and 76% removal were recorded at 40 min for PL powder when treated with the same concentrations, respectively. While seemed adequate for the PFO kinetics model (Fig. 3A), the adsorption data could not be fulfilled perfectly in its linear form Eq. (5). This was verified for the adsorption data of MB onto PL and PR (Fig. 3B). On the other hand, the PSO kinetics model (Fig. 3C) appeared more appropriate for fitting the adsorption data of MB both onto PL and PR (R^2^ ≈ 1). The WM intra-particle diffusion model (Fig. 3D) did not perform well like the PSO model but was better than the PFO model with R^2^ = 0.79. This diminishes the significance of MB diffusion within the PL and PR and dedicates the adsorption kinetics principally to the surface MB–adsorbent interaction. Interestingly, the PSO model predicted that the amounts of MB that would adhere to the PL and PR at equilibrium would be around 10.63 and 15.02 mg/g, respectively when the initial concentration of MB was 0.1 g/L, which was exactly in line with the corresponding experimental data, ${q}_{e,exp}$, (10.63 mg/g for PL and 15.19 mg/g for PR). The slope and intercept of Fig. 3C were used to determine the rate constants, which were found to be 0.47 and 0.07 mg/g/min (Table 2). Consequently, the adsorption rates, $h$, of 52.66 and 15.78 mg/g/min were assigned for the adsorption of MB onto PL and PR, respectively.

**Table 1:** Regression coefficients (R^2^), equilibrium adsorption capacities (${\mathrm{q}}_{\mathrm{e}}$) of MB (mg/g) and the rate constants (${\mathrm{k}}_{1}$) of the PFO kinetics model as obtained from the regression of Fig. 3A using the exponential equation: ${\mathrm{q}}_{\mathrm{t}}={\mathrm{q}}_{\mathrm{e}}\left[1-\mathrm{exp}\left(-{\mathrm{k}}_{1}\mathrm{t}\right)\right]$.

Leaves			Rhizome		
R^2^	${\mathrm{q}}_{\mathrm{e}}$(mg/g)	${\mathrm{k}}_{1}$(min^−1^)	R^2^	${\mathrm{q}}_{\mathrm{e}}$(mg/g)	${\mathrm{k}}_{1}$(min^−1^)
0.999	10.537	0.775	0.988	14.499	0.493

**Table 2:** A summary of kinetic data obtained from Fig. 3.

Kinetics model	Leaves			Rhizome		
	R^2^	Slope	Intercept	R^2^	Slope	Intercept
PFO	0.58	− 0.06	− 0.26	0.81	9.5299459 × 10^–3^	0.32
PSO	1.00	0.09	0.01	1.00	0.07	0.05
WM	0.79	6.80	− 11.61	0.70	7.34	− 13.17

### Plant dosage

**Figure 4:**
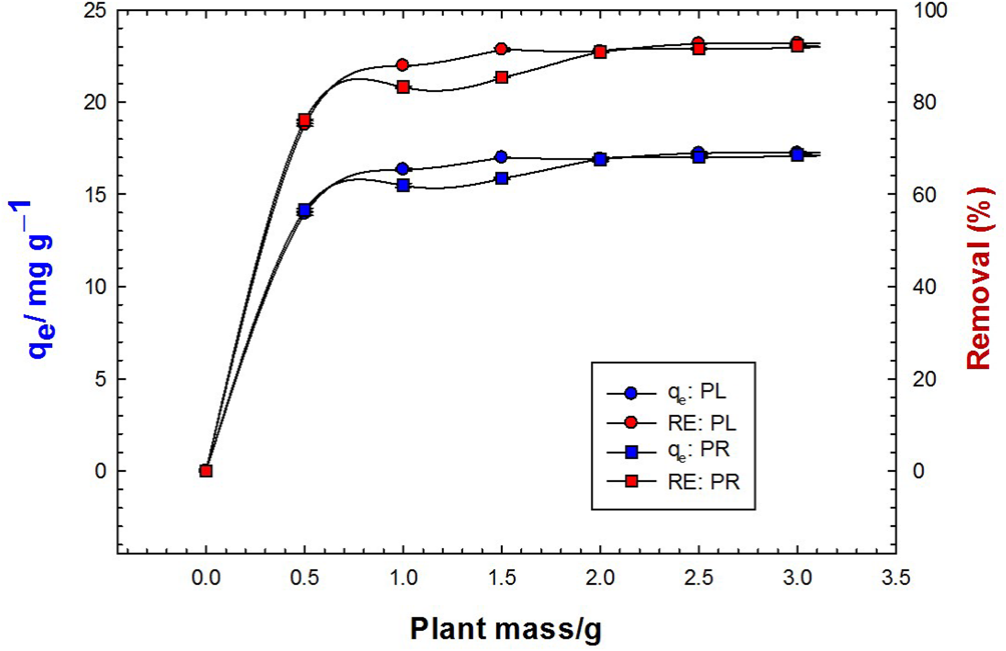
Effect of plant dry biomass on the adsorption capacity (blue color) and removal efficiency (red color) of 0.1 g/L MB (natural pH) onto PL (circles) and PR (squares).

The impact of the plant dosage on the adsorption uptake and efficiency is shown in Fig. 4. It is apparent that the MB adsorption increased rapidly with the plant dosage, because of increasing the adsorption sites available for the MB removal. Interestingly, 0.5 g PR powder induced ca. 76 and 73% removal efficiencies when treated with 0.1 g/L and 0.4 g/L of MB-containing solution, respectively. On the other hand, the same mass of PL powder recorded 75 and 70% removal efficiencies for the same solutions, respectively. Adsorption efficiencies up to 92.8 and 92.5% could be verified for the adsorption of MB onto the PL and PR, respectively, at higher doses of plant powder. Nonetheless, the influence of the plant mass on the adsorption capacity and efficiency of MB was minor within the range of 1 g/100 mL (10 g/L) to 2 g/100 mL (20 g/L) of PL and PR biomass, respectively. These results led us to believe that 10 g/L of PL and PR was the optimal dosage while also attempting to limit the variables.

### Effect of MB dye initial concentration

**Figure 5:**
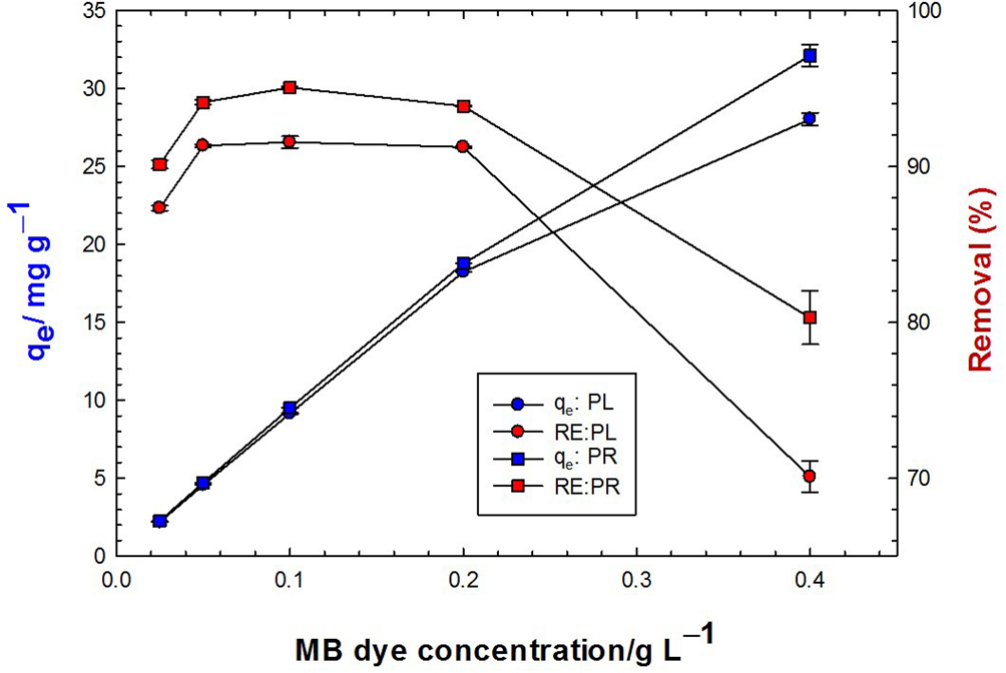
Effect of adsorbate (MB) initial concentration on the adsorption capacity (blue color) and removal efficiency (red color) onto 10 g/L PL (circles) and PR (squares) at natural pH.

The investigation continued to analyze the role of the initial adsorbate concentration where several samples of MB with concentrations from 0.025 to 0.400 g/L were tested while maintaining the optimum adsorbent dosage of 10 g/L. From an industrial perspective, this analysis is highly important due to the presence of MB in variable concentrations in the industrial streams. In general, the removal tendency of plant materials was higher when treated with diluted MB solution. This ability decreased by increasing adsorbate concentration, but the actual amount of pollutant adsorbed per unit mass of adsorbent increased with increased adsorbate concentrations in the test solution. As shown in Fig. 5 a sample with 0.025 g/L MB showed removal efficiencies of 94 and 92% when treated with 1 g of PR and PL powder, respectively, while the same concentration amounted to 98% removal when treated with 3 g of both plant materials. On the other hand, a sample with 4 g/L MB attained removal efficiencies up to 89 and 87% when treated with 1 g and 96 and 98% when treated with 3 g of PR and PL powders, respectively.

Figure 5 shows a linear increase of the equilibrium adsorption uptake of MB with the MB concentration up to 0.2 g/L with a minor distinction for PR over PL. This linear relationship continued up to 0.4 g/L MB but with lower slopes. The MB removal efficiency, on the other hand, depended largely on the initial MB concentration, and the highest efficiencies (up to 97 and 95% for PL and PR, respectively) were reported for intermediate concentrations (from 0.05 to 0.2 g/L MB). It is worth mentioning that the increase in the MB concentration to 0.4 g/L did not associate with a parallel and equivalent increase in the adsorption uptake, and hence, the removal efficiency decreased remarkably.

### Adsorption isotherms

Four adsorption models—Freundlich, Langmuir, Temkin, and D-R isotherms—were used to simulate the interaction of MB dye at the surface of the PL and PR biomass and to determine their maximum adsorption capacities for the removal of MB dye.

**Figure 6:**
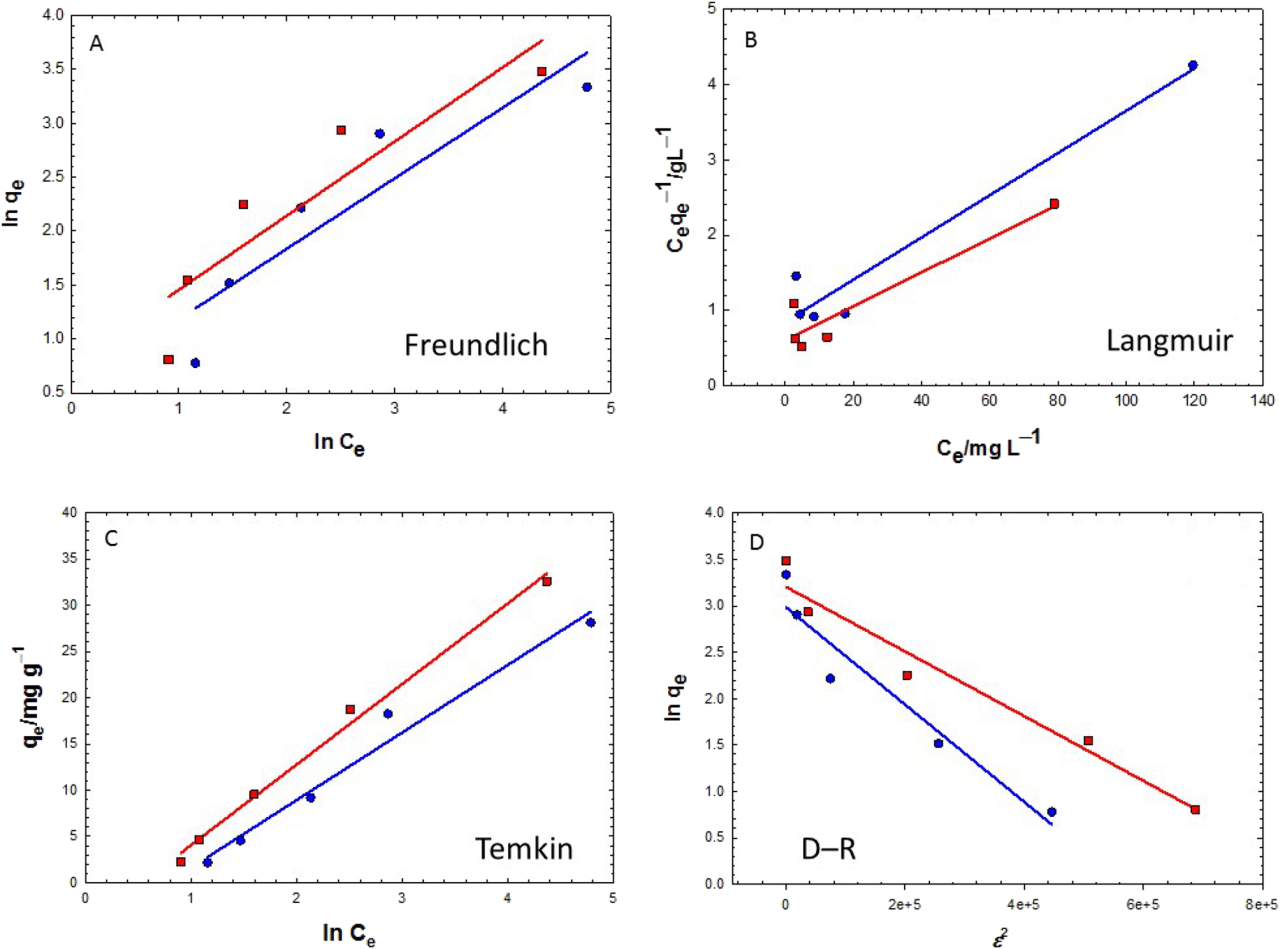
Fitting the experimental adsorption data of MB according to the (**A**) Freundlich, (**B**) Langmuir, (**C**) Temkin, and (**D**) D–R adsorption isotherms using 10 g/L PL (blue circles) and PR (red squares) at natural pH.

Figure 6A shows the ${q}_{e}$ and ${C}_{e}$ correlation according to the Freundlich model Eq. (9) and evaluates the ${k}_{F}$, *n* and 1/*n* constants for the adsorption of MB dye onto the PL and PR biomass at room temperature. The R^2^ values of both plots in Fig. 6A were below 0.85, suggesting poor fittings of experimental data according to the Freundlich isotherm (Table 3). The values of ${k}_{F}$, *n* and 1/*n* were 1.70, 1.53, and 0.65, respectively, for PL and were 1.99, 1.30, and 0.77, respectively, for PR biomass. It is observed that 1/*n* value was low (< 1) while ${k}_{F}$ value for the adsorption of MB onto the PR was high.

**Table 3:** A summary of adsorption isotherms data obtained from Fig. 6.

Adsorption isotherms	Leaves			Rhizome		
	R^2^	Slope	Intercept	R^2^	Slope	Intercept
Freundlich	0.84	0.65	0.53	0.83	0.69	0.77
Langmuir	0.95	0.03	0.85	0.88	0.02	0.61
Temkin	0.98	7.31	− 5.67	0.99	8.71	− 4.64
D–R	0.93	− 5.2493089 × 10^−6^	2.98	0.96	− 3.4881390 × 10^−6^	3.21

On the other hand, to examine the Langmuir model’s applicability to the experimental data, Fig. 6B was plotted for $\frac{{C}_{e}}{{q}_{e}}$ vs. ${C}_{e}$ (Eq. 10). It is interesting to note that the adsorption of MB onto the PL and PR was associated with regression factors (R^2^) higher than those found for the Freundlich model (see Table 3). The maximum monolayer adsorption capacities, ${q}_{m}$, for the MB adsorption onto PL and PR were 35.7 and 212.9 mg/g, while ${K}_{L}$ were 0.0329 and 0.0279 L/mg, respectively. According to the Langmuir model, the adsorption feasibility for different adsorbate concentrations is assessed by the separation factor (R_L_) which is a dimensionless parameter as seen in Eq. (15)^57^.

15 $${R}_{L}=\frac{1}{{1+K}_{L}{C}_{0}}.$$

For ${R}_{L}>1$, the adsorption is unfavorable, for ${R}_{L}=1$, the adsorption is linear, for ${0<R}_{L}<1$, the adsorption is favorable, and for ${R}_{L}=0$, the adsorption is irreversible. Applying these criteria to the experimental data of MB adsorption onto the PL and PR using an initial MB dye concentration, C_0_, of 100 mg/L indicates that these adsorption processes are favorable.

The Temkin model Eq. (11) was next applied to test the ${q}_{e}$ vs. ${C}_{e}$ correlation as shown in Fig. 6C. It is revealed that the best correlation for the experimental data was obtained with this model, as indicated by the R^2^ (> 0.97) value (Table 3). For the adsorption of MB dye onto the PL and PR, the Temkin constants $b$ were computed to be 333.18 and 279.52 J/mol while the constants $A$ were 0.461 and 0.587 L/g, respectively (Fig. 6C).

Finally, the experimental data were tested for fitting according to the D − R isotherm (Eqs. 12–14). A simple plot of $ln{q}_{e}$ vs. ${\varepsilon }^{2}$ (Fig. 6D) could predict $\beta$ and ${q}_{m}$ from the slope and intercept, respectively (Table 3). A substitution in Eq. (14) could evaluate $E$. Regression factors higher than 0.92 were obtained for modeling the adsorption data of MB dye onto the PL and PR according to this model. For the adsorption of MB dye onto the PL and PR, ${q}_{m}$ were calculated to be 19.78 and 24.67 mg/g while $E$ were 0.31 and 0.38 kJ/mol, respectively (Fig. 6D). The results demonstrated that the model anticipated a higher ${q}_{m}$ for PR than PL for the removal of MB dye, which is in line with the experimental data. Additionally, based on the low E values, it determined that physisorption mechanisms were responsible for the removal of MB from both the PL and the PR dry biomass.

### Effect of shaking time

**Figure 7:**
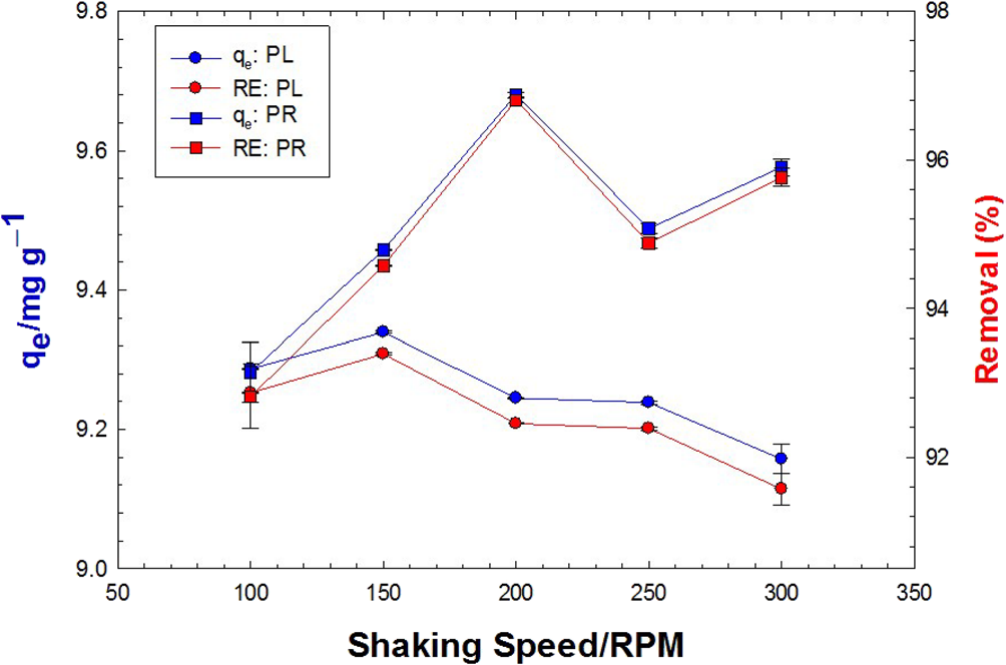
Effect of shaking speed on the adsorption capacity (blue color) and removal efficiency (red color) of 0.1 g/L MB using 10 g/L PL (circles) and PR (squares) at natural pH.

In the present study, the impact of shaking speed showed a minor effect on the adsorption of pollutants on PR and PL powders. When the shaking speed was increased from 100 to 300 rpm, 1 g of PR powder achieved up to 96% removal efficiency, and 3 g of the same plant material achieved up to 98% efficiency. Besides, the PL powder showed about 92 and 98% removal efficiency under the same conditions, respectively. The results on the effect of shaking speed between 100 and 300 RPM on the adsorption capacity and removal efficiency of 0.1 g/L MB using 10 g/L are shown in (Fig. 7). The optimization of the adsorption process at 150 RPM with an adsorption capacity of 9.34 mg/g and efficiency of 93.4% in PL was obtained. On the other hand, 200 RPM was optimized with an adsorption capacity of 9.68 mg/g and an efficiency of 96.8% for PR. It remains to visualize the adsorption proceeding in a reversible (adsorption/desorption) mode. Accelerated shaking rates may inspire different impacts on the rates of both the adsorption and desorption processes. Mechanically, high shaking rates can overcome the physical attraction forces between the sorbate and sorbent, enhancing the return of MB dye back to the solution and decreasing the adsorption uptake. That is essentially what was seen at shaking rates higher than 150 and 200 RPM in PL and PR, respectively. However, the higher tolerance of the MB-PR bonding for dissociation (desorption) early at 150 RPM was likely caused by the higher adsorption energy of MB onto PR (0.38 kJ/mol) than that of PL (0.31 kJ/mol).

### Effect of temperature

**Figure 8:**
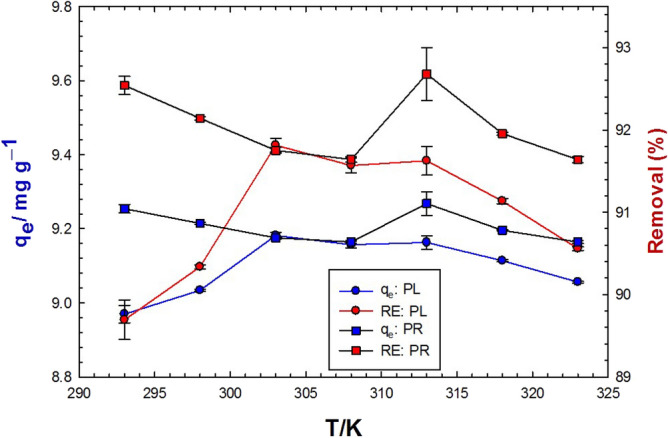
Effect of temperature on the adsorption capacity (blue color) and removal efficiency (red color) of 0.1 g/L MB using 10 g/L PL (circles) and PR (squares) at natural pH.

Figure 8 showed that the equilibrium adsorption uptake of MB dye onto PL increased slightly from 20 to 30 °C then became approximately constant up to 40 °C, and then steadily decreased to 50 °C. The trend of MB dye adsorption onto PR was a little different as the equilibrium adsorption uptake decreased directly from 20 to 33 °C and increased again at 40 °C, and then continued decreasing up to 50 °C. The removal efficiencies did not change largely to the same extent as the adsorption uptake and the highest efficiencies were recorded at 30 and 40 °C on PL (91.9%) and PR (93.3%), respectively. The minor variation in the heat of adsorption of MB onto PL and PR, along with the geometrical differences between PL and PR, might be the reason for the observed behavior.

### Effect of pH

**Figure 9:**
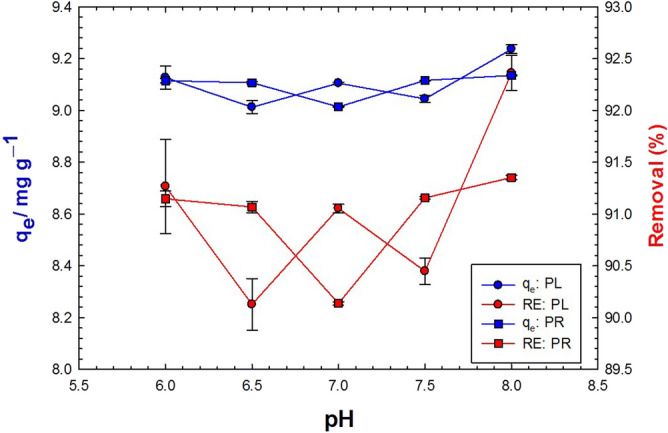
Effect of pH on the adsorption capacity (blue color) and removal efficiency (red color) of 0.1 g/L MB using 10 g/L PL (circles) and PR (squares) at natural pH.

The impact of pH (6–8) on the adsorption process of MB dye was quite difficult (Fig. 9). This remained true for both PL and PR, showing that this pH range—close to neutrality—almost results in uptakes and efficiencies that were extremely similar. The removal efficiencies (94% using 1.0 g and 98% using 3.0 g of PR powder) were enhanced in the slightly acidic medium (pH 6). On the other hand, the PL powder showed the best removal efficiencies (93% and 98% using the same weights, respectively) in the slightly basic medium (pH 8).

## Discussion

### Materials characterization

We are the first to report the functional group analysis of the PL and PR biomass of *Vossia* cuspidata. The major broad peaks at 3417 and 3424 cm^−1^ for PL and PR, respectively, belong mostly to the –NH/–OH overlapped stretching vibration, as commonly assigned in similar plants^66–68^. The other major peaks at 2924, 1646, and 1050 cm^−1^ for PL (Fig. 1A), and at 2925, 1639, and 1054 cm^−1^ for PR (Fig. 1B) can likely refer to the (C–H) asymmetric stretching, overlapped C=N stretching and N–H bending, and C–O–C and C–N overlapped stretching, respectively^68–71^. On the other hand, the minor absorption peaks of PL (Fig. 1A) were assigned to the C=O stretching of aldehyde (1728 cm^−1^), C=C stretching of imine/oxime (1646 cm^−1^), N–O stretching (1518 cm^−1^), C=C side ring stretching (1428 cm^−1^), –CH_2_ or –CH_3_ stretching (1381 cm^−1^), –C–N stretching (1250 cm^−1^), –C–O stretching of tertiary alcohol (1158 cm^−1^), C=C bending (902 cm^−1^), C–H bending (598 cm^−1^), Si–O–Si stretching (467 cm^−1^), and Si–O stretching (429 cm^−1^), respectively^68,72–74^. On the other hand, the minor peaks of PR biomass (Fig. 1B) were assigned similarly to the C=O stretching of aldehyde (1721 cm^−1^), N–O stretching (1513 cm^−1^), C=C side ring stretching (1426 cm^−1^), –C–N stretching (1254 cm^−1^), –C–O stretching of tertiary alcohol (1157 cm^−1^), C–H bending (605 cm^−1^)^68,72^. The loading of MB dye onto the PL biomass did not inspire great shifts in the peak positions, in contrast to the case of PR biomass. It is worth mentioning that the FTIR spectrum of the MB dye itself depicted very similar absorptions to most of those of unloaded PL and PR^68^. Further chemical analysis is required to understand the nature of the interaction between MB dye and PL and PR biomasses.

The surface morphology (Fig. 2) of the *V. cuspidata* biomass (PL and PR) appeared porous and bundled with dispersed microcavities, which is a favorable structure for the pore-filling adsorption mechanism. The parenchyma and aerenchyma as well as xylem vessels in both plant materials showed an irregular porous morphology at the plant surface. The white debris that were appeared after loading the PL and PR surface with MB (Fig. 2C,D) can likely return to MB.

### Adsorption kinetics

In this study, the appropriateness of the PSO kinetic model was confirmed for fitting the adsorption of MB dye onto the dry powder of *V. cuspidata*. The same model was previously recommended for the adsorption of organic dyes by plant dry biomasses^18,75,76^. The equilibrium adsorption uptakes, ${q}_{e}$, of PR and PL were 14.50 and 10.54 mg/g. While removal efficiencies of 97 and 94% for MB were obtained onto the PR and PL powders (1 g/100 mL) after 35 and 40 min, respectively. These values stand among the highest reported values for MB from aqueous contaminated streams (Table 4)^77–95^.

**Table 4:** Adsorption capacity and removal (%) of MB.

Adsorbent	Adsorption capacity, mg/g	Removal (%)	References
Activated carbon	9.81	98.5	^80^
Hazelnut shell-activated carbon 750 °C	8.82	–	^81^
Coir pith carbon	5.87	97–100	^82^
Apricot stones-activated carbon 750 °C	4.11	–	^81^
Walnut shell-activated carbon 750 °C	3.53	–	^81^
Almond shell-activated carbon 750 °C	1.33	–	^81^
Fir wood based activated carbon	1.21	–	^83^
Corncob based activated carbon	0.84	–	^84^
Calcined pure kaolin	8.88	~ 100	^85^
Calcined raw kaolin	7.59	–	^85^
Clay	6.3	–	^86^
Glass wool	2.24		^87^
*Posidonia oceanica* (L.) fibres	5.56	37–100	^88^
*Caulerpa racemosa* var. *cylindracea*	5.23	81–98	^89^
Living biomass	1.17	–	^90^
Raw beech sawdust	9.78	–	^91^
Coarse grinded wheat straw	3.82	–	^92^
Neem (*Azadirachta indica*) leaf powder	3.67	85–95	^93^
Fine grinded wheat straw	2.23	–	^92^
Fly ash	3.07	96	^80^
Chrome sludge	0.51	15–40	^94^
Cow dung ash	5.31	–	^95^
*Vossia cuspidata* leaves biomass	10.5	94	This study
*Vossia cuspidata* rhizome biomass	14.5	97	This study

The rapid adsorption rate of any chemical may be due to the presence of a substantial number of unoccupied adsorption sites existing on the adsorbent surface^96^. The presence of MB in contact with the powder of *V. cuspidata* rhizome and leaves causes a sudden accumulation of dye on the surface of the adsorbent. With time, these sites become occupied by MB which may produce a repulsive force between the molecules of dye on the adsorbent surface as reported by Mahmoodi et al.^97^. At higher dye concentrations, the number of dye molecules competing for the available sites on the surface of the adsorbent is large which may explain the faster equilibrium time. This rapid adsorption time is recommended and highly desirable in the application of this technology. When employing activated carbon made from *Ficus carica* wood and modified oak residue, the equilibrium contact time of MB was reached in 90 min and 120 min, respectively^98,99^. It was observed that the equilibrium times for MB removal onto *V. cuspidata* were shorter, which indicates better adsorbent materials.

### Plant dosage

Studying the adsorbent dose used in the adsorption process is a vital factor, as it determines the equilibrium conditions between the adsorbent and pollutants in the reaction system that can be used to evaluate the ecological cost-benefits during the treatment of polluted habitats^100^. Increasing the adsorbent dose causes an increase in the contact surface area with much more active sites for binding with MB. Many factors may contribute to the higher removal capacity, including solute availability, electrostatic interactions, interference between binding sites, and lower mixing at high biomass densities^101^. The findings of the present study agreed with that reported by Esmaeili and Foroutan^102^.

### Effect of MB dye initial concentration

At constant adsorbent dose, *V. cuspidata* powders showed different removal rates when treated with different dye concentrations. This phenomenon is very much related to diffusion constraints. If the MB concentration is low, the ratio of MB to the number of available active adsorption sites is small, which encounters the mass transfer of MB from the aqueous to the PL and PR surface to yield low adsorption uptakes. On the other hand, higher initial MB concentrations may overcome the diffusion limitations and support higher MB fluxes to the plant surface, increasing the adsorption uptakes^58–60^. Concerning constant biomass weight, increasing dye concentration (up to 4 g/L) resulted in a slight decrease in removal capacity, which indicates the high ability of the plant biomass for adsorption wherever the position of adsorbent from the source point of pollution. Regarding the adsorption process, the quantity of adsorbed dyes increased as the initial dye concentration increased. The increase in the concentration gradient with increasing dye initial concentration could explain this phenomenon^103^.

### Adsorption isotherms

On the other hand, the Freundlich adsorption isotherm model had low (< 1) 1/*n* values but high ${k}_{F}$ values for the adsorption of MB onto the PL and PR. Small numerical values for 1/*n* (< 1) not only confirm the favorable adsorption of MB onto the PL and PR but also indicate the nature of physical bonding between MB and the adsorbent^54^. The higher ${k}_{F}$ for the adsorption of MB onto the PR indicated a corresponding higher adsorption capacity. Likewise, a higher *n* value for the MB adsorption onto PL suggested a more favorable adsorption than onto PR. The Temkin model showed the best correlation for the experimental data compared to other tested models. Consequently, we can suggest the Temkin model for the adsorption of the MB dye onto the PL and PR. In a previous investigation, the same model was suggested (R^2^ = 0.95) for the adsorption of MB onto Miswak (*Salvadora persica*) leaves in an alkaline solution with an adsorption capacity of 200 mg/g^104^. Al-Ghouti and Al-Absi investigated the cationic methylene blue dye's adsorption and thermodynamic effects on cellulosic olive stone biomass^18^. They found that the green olive stones obtained the highest R^2^ value with the Temkin isotherm model, whereas the black olive stones showed the highest R^2^ value with the Langmuir isotherm model.

The same results were reported by Amin et al*.* for the batch adsorption of crystal violet and methylene blue dyes from an aqueous solution using *Eucalyptus camdulensis* biochar^76^. The Temkin isotherm was nearly a perfect fit to the adsorption data of the MB dye, with an R^2^ value close to unity (1.0). This significant correlation shows that the MB dye was adsorbed in a heterogeneous manner with a homogeneous distribution of binding energies.

**Figure 10:**
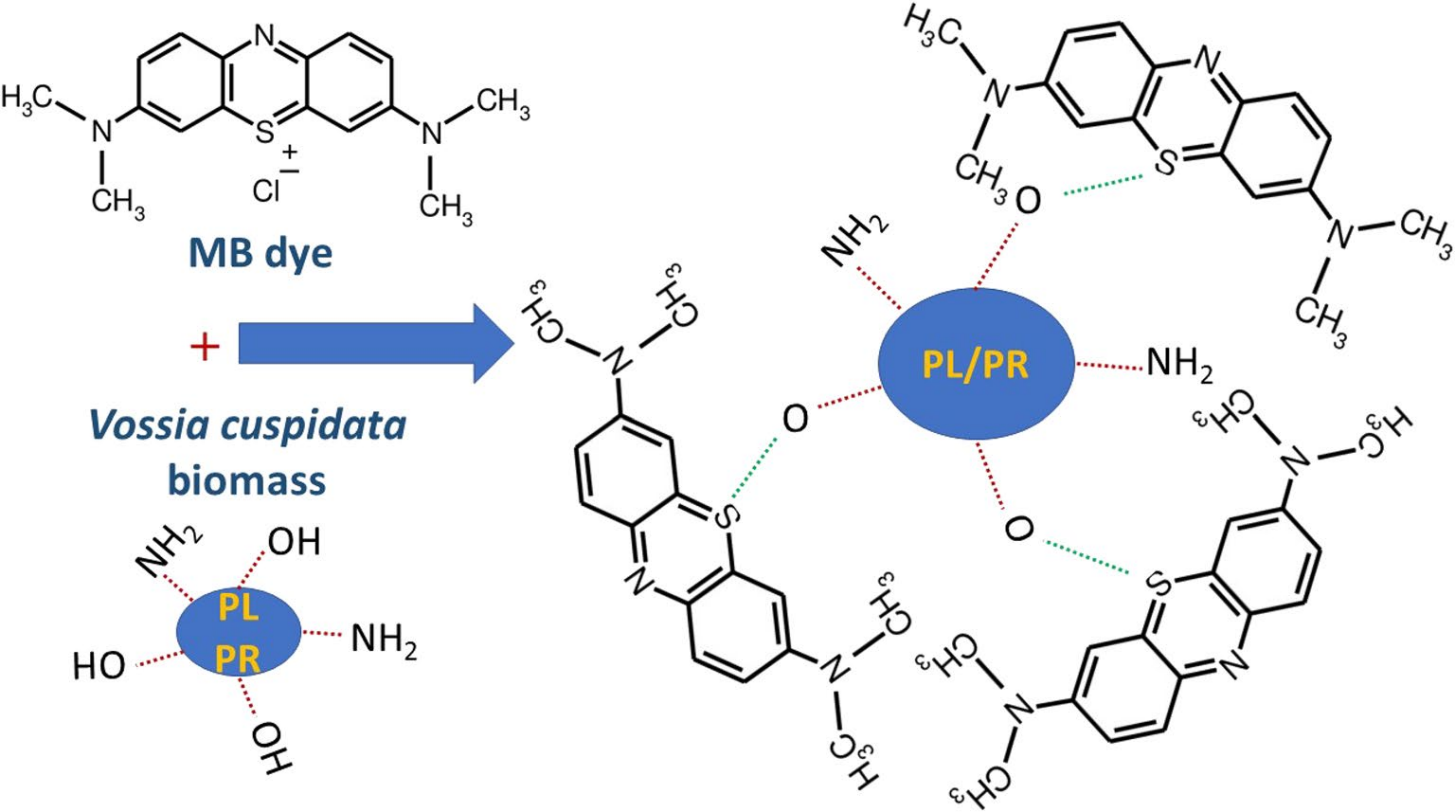
Plausible adsorption mechanism for the removal of MB by *V. cuspidata* biomass. The green dotted lines represent the physical dipole–dipole interactions between MB and the plant.

The nature of adsorbent–adsorbate interaction can be predicted from data obtained from the investigated adsorption isotherms in this study. The Temkin constant $b$ that is related to the adsorption energy was 333.18 and 279.52 J/mol for the adsorption of MB dye onto the PL and PR. The average adsorption energy, $E$, for the adsorption of MB dye onto the PL and PR was estimated from the D–R isotherm as 0.31 and 0.38 kJ/mol, respectively, which agreed to a great extent with the Temkin constant. These findings came consistent with the values of 1/*n* (0.65 and 0.77 for the adsorption of MB onto PL and PR biomass, respectively) that were computed by the Freundlich adsorption isotherm model to indicate the nature of physical bonding between MB and the adsorbent^54^. One of the common physical bonding is the dipole–dipole interaction which is likely to prevail due to the existence of positive centers at the sulfur atom of MB and positive centers at the oxygen atoms of the hydroxyl groups that enrich the plant surface (as evidenced from IR spectra). The schematic in Fig. 10 illustrates this sort of attractions. More chemical analysis and materials characterizations are required to specifically assign the sort of the prevalent physical interaction.

### Effect of shaking time

To expedite the mass transfer of MB to the sorbent surface, the influence of shaking rate, a hydrodynamic parameter, was then examined. Noubactep et al. investigated previously the impact of shaking rate between 0 and 300 min^−1^ on the adsorption of MB onto scrap iron (Fe^0^), granular activated carbon (GAC), and deep-sea manganese nodules (MnO_2_)^24^. While non-shaken experiments lasted for up to 50 days, shaken experiments were executed in less than a day. Their results suggested 50 min^−1^ as the optimum shaking rate for the facilitated mass transfer and the highest MB discoloration with no damage or dissolution of the sorbent material. Shaking is an essential factor that affects the bombardments between adsorbate and active sites on adsorbent. Physically, there are two types of mass transfer resistances: external and internal. The external one is due to the external diffusion between solution and adsorbent particles, while the internal one is due to the diffusion within the particles. Changing the shaking speed causes not much change in removal efficiency^75^. This is an indicator of the negligible effect of external mass transfer resistance (external diffusion) at these shaking speeds.

### Effect of temperature

One of the most important parameters influencing the rates of adsorption and desorption of metal ions and organic dyes is the operating temperature. High temperatures kinetically speed up both, facilitating attaining the equilibrium quickly at high equilibrium constants. The mass transfer of the adsorbate from/to bulk solution is also enhanced at elevated temperatures. In addition, the adsorption/desorption processes follow the equilibrium constraints, and raising the temperature would favor one direction at the expense of the other based on Le Chatelier’s principle. Thermodynamically, adsorption processes are always exothermic, which necessitates boosting the desorption process at elevated temperatures, decreasing the adsorption uptake. It is also known that by increasing the temperature, the viscosity of the adsorbate solution decreased, and the movement of dye particles increased. The slight change in the “high” adsorption rate within the wide range of tested temperatures (20–50 °C), indicates the reduced economic cost of the adsorption process requirements^103^. Accordingly, *V. cuspidata* adsorbent biomass can remove considerable amounts of dyes at a variety of temperatures which represents an effective adsorptive material.

### Effect of pH

The hydrogen ion (pH) concentration of any solution has a considerable effect on the adsorption process. The pH affects the ionization of the dye molecule as well as the charge of functional groups on the adsorbent materials^105^. Among the studied range of hydrogen ion concentration, our results in Fig. 9 showed that the adsorptive capacity of the rhizome powder was higher in the slightly acidic medium (pH 6), while leaf powder showed higher capacity in the slightly basic medium (pH 8). The difference in the favorable adsorption pH values between rhizome and leaves powders of *V. cuspidata* is likely due to the difference in chemical composition and active site charges of each adsorbent material. In an acidic medium, the positively charged adsorbent sites are increased and become more than the negatively charged sites. On the contrary, in the basic medium, the numbers of negatively charged sites are more than the positive ones^106^. The normal pH of natural environments varies from slightly acidic to slightly alkaline media, which provides ideal adsorption conditions for *V. cuspidata* powders^107^.

## Conclusion

The dry biomass of *V. cuspidata* rhizomes including aerial stems and leaves could be used as efficient materials in the phytoremediation of MB dye from industrial sources that contaminates water bodies. A removal efficiency up to 97% was obtained in 35 min for 0.1 g/L MB. Increasing the plant dosage increased the adsorption uptake with high dependence on the initial MB concentration. The experimental data could perfectly be fitted in the Temkin isotherm model where the energy parameters suggested the physical interaction between the plant biomass and MB dye. Further research should be done on the optimal settings and packing characteristics for the plant powders according to industrial purposes.

## Data Availability

All data generated during this study are included in this published article.
